# Study of the Correlation between Streaming Video Platform Content on Food Production Processes and the Behavioral Intentions of Generation Z

**DOI:** 10.3390/foods13101537

**Published:** 2024-05-15

**Authors:** Xi-Yu Zhang, Ching-Tzu Chao, Yi-Tse Chiu, Han-Shen Chen

**Affiliations:** 1 Department of Accounting, School of Business, Nanjing University, Nanjing 210093, China; xiyuz3@163.com; 2 Department of Health Industry Technology Management, Chung Shan Medical University, Taichung 40201, Taiwan; tiffany891224@gmail.com (C.-T.C.); jimmy060909@gmail.com (Y.-T.C.); 3 Department of Medical Management, Chung Shan Medical University Hospital, Taichung 40201, Taiwan

**Keywords:** food processing, streaming service, theory of planned behavior (TPB), perceived trust, perceived risk

## Abstract

With an estimated 2.4 million cases of foodborne illnesses recorded annually in the UK alone, food safety has become a paramount concern among stakeholders. Modern technology has positioned streaming platforms as pivotal conduits for disseminating information. Channels such as YouTube offer detailed recordings of the food production process, granting consumers extensive visibility of the food journey from farm to table. This increased transparency not only promotes vigilant monitoring of food safety practices but also solicits consumer feedback regarding the public exposure to food processing videos. Based on the Theory of Planned Behavior (TPB), this study augments its framework with constructs, such as perceived trust, perceived risk, community experience, and brand identity, to evaluate Taiwan’s Generation Z consumer behavioral intentions. With 226 valid responses amassed, structural equation modeling facilitated elucidation of the relationships among the constructs. This analysis yielded three salient insights. First, Generation Z’s engagement with food processing videos on streaming platforms is positively correlated with their subsequent purchasing behavior. Second, enriched community experience was correlated with strengthened brand identification. Third, both perceived trust and perceived risk had a constructive impact on behavioral intentions within Gen Z’s demographic data. Based on these outcomes, food industry enterprises should proactively develop and bolster community experiential value, thereby encouraging streaming platform users to transform into brand consumers and advocates.

## 1. Introduction

The backdrop of heightened concern over food safety, underscored by the 2.4 million annual cases of foodborne illnesses in the UK alone [1], spotlights the need for more effective food safety measures and consumer education. Incidents of microbial contamination from *Salmonella* and *E. coli* in poultry in Nigerian markets [2] to Listeria in European-imported artisanal goat cheese [3], as well as the life-threatening instance of bongkrekic acid poisoning in Taiwan, demonstrate crucial points where knowledge gaps can have dire consequences. These events not only reveal inherent flaws in food production and safety protocols, but also highlight a broader issue: the widespread lack of knowledge among consumers regarding how their food is produced, processed, and safeguarded.

Such an insufficient understanding can lead to misinformed choices at the consumer level, contributing to the spread of myths about food production practices. The complexities of food safety from “farm to table” underline the imperative for not just stringent safety measures but also comprehensive consumer education [4,5]. A more informed consumer base is essential to effectively navigating these challenges, which is the focal point of this work. By bridging the knowledge gap, this research seeks to illuminate the ways in which an improved understanding of food production processes can impact consumer choice, dispel misconceptions, and ultimately encourage safer and more conscious food consumption practices.

Technological innovation has precipitated the rise of streaming services as an essential medium for disseminating information. Within this landscape, YouTube is a predominant video-sharing platform, with over two billion daily views and 95% of Internet users visiting the site [6]. This extensive reach makes it an optimal conduit to promote transparency. Projection of the food-making process onto such a platform does not solely provide the product with increased exposure; it also engenders deeper consumer understanding and trust in the product. De Blanes Sebastián and de Matías Batalla [7] indicate that streaming media have led to significant shifts in consumer perceptions and behaviors. Ding et al. [8] demonstrated the incremental value of live streams of restaurant kitchens to online food delivery (OFD) services, where the broadcasting of food safety information underpins user welfare benefits that far outweigh operational costs, fostering a positive consumer perception of food safety practices. According to Yang [9], respondents’ most frequently purchased items through live-stream shopping are food products, followed by beauty products. Therefore, given the economic significance of online videos, understanding video consumption behavior is crucial for video platforms and marketers [10].

Simultaneously, there is a discernible shift in market dynamics with Generation Z users, who are active and engaged YouTube consumers, demonstrating prolific rates of usage, channel subscriptions, content preference, and interactive behaviors, such as sharing and commenting, positioning them as a prime demographic for targeted influencer marketing strategies [11]. Evidence suggests that digital marketing through social media, websites, and blogs can significantly influence Generation Z’s purchasing choices [11]. This paradigm shift elevates the importance of developing tailored content that resonates with the preferences and behavioral patterns of this generation.

In leveraging streaming platforms to document the food manufacturing journey, stakeholders do more than capture procedural narratives, opening a window for the public scrutiny of sanitation conditions, quality assurance measures, workforce hygiene, operational integrity, and storage and transportation logistics, ensuring that consumers can validate the quality and safety of food products throughout their lifecycle. However, the influence of such transparent insights on consumer purchasing behavior merits closer examination. Historical shortcomings include the omission of direct purchase links in food-related content on these platforms and the question of the actual conversion rate of viewers to consumers. Given these gaps in research, this study is strategically positioned to offer substantial contributions through an in-depth analysis.

The primary aim of this study is to investigate the comprehensive impact of food processing videos on digital platforms in shaping Taiwanese consumers’ perceptions of food production transparency and its subsequent effects on their purchasing behaviors. Specifically, this study focuses on understanding how such content influences consumer trust in food safety and quality, the processing and reception of this information by Generation Z consumers via social media, and its broader implications for consumption patterns. Additionally, by employing the Theory of Planned Behavior (TPB), this study explores the role of perceived trust, risk, community experience, and brand identity in determining acceptance of and behavioral intentions towards food production among Taiwanese consumers. By achieving these objectives, this study seeks to enhance the effectiveness of digital media in communicating about food safety and to identify ways to increase consumers’ understanding of food production processes, ultimately guiding their purchasing decisions. This study provides insights for the food industry to optimize marketing strategies and help consumers make informed food choices.

## 2. Literature Review and Hypothesis Development

### 2.1. Theory of Planned Behavior

To explore the realm of consumer behavior towards transparent food production processes, this study hinges on the Theory of Planned Behavior (TPB) as the foundational framework. The TPB, propounded by Ajzen [12], is instrumental in predicting and explaining behavioral intentions that lead to specific actions within contextual constraints. Central to this theory are three determinative elements: attitude, subjective norms, and perceived behavioral control. These elements have consistently been empirically validated across a plethora of studies, underscoring their significance in predicting behavioral intentions [13,14,15,16]. The underlying components of this theory are as follows.

#### 2.1.1. Behavioral Intentions

It is pivotal to delineate ‘behavioral intentions’ within the TPB framework, as it pertains to this research. Behavioral intentions represent the predispositional strength driving an individual towards a specific behavior, encapsulating the motivational factors that influence the execution of a behavior [17]. This conceptualization posits intentions as predictors of actual behavior, subject to the influence of attitudes, subjective norms, and perceived behavioral control [18].

#### 2.1.2. Attitude

An individual’s attitude epitomizes the favorable or unfavorable evaluation of a behavior [19]. Evidence suggests that attitudes predict subsequent behavior with the degree of influence of conviction of attitudes, individual personality traits, and sociocultural background [20]. Studies have established that various factors within live streaming, such as streamer attractiveness, social interaction, and information quality, significantly influence viewers’ cognitive and affective states, subsequently altering their conduct in a live-commerce context [21]. Moreover, the congruence of influencers and product origin with consumer expectations can impact perceived credibility and startup attitudes towards content, thus affecting behavior [22]. Accordingly, we propose the following hypothesis:

**H1a:**  *Consumers’ attitudes towards transparent food production processes positively influence their behavioral intentions.*

#### 2.1.3. Subjective Norms

Subjective norms encompass the perceived social pressure to perform or refrain from a behavior, which is determined by an individual’s belief about what ‘important others’ may think [19]. Such norms not only serve as precursors to personal norms but also considerably shape consumer behavior [23]. The likelihood of Brazilian consumers using food labels for healthy eating choices has been linked to peer influence [24]. Urban consumers’ buying decisions are influenced by external factors, such as culture, social class, personal influence, family, and situation, as well as internal factors, such as knowledge, attitude, personality, lifestyle, and valence [25].

Findings by Xie and Madni [26] suggest that social media usage positively impacts green consumption among the younger generation in China, with subjective norms and perceived green values playing a pivotal mediating role. This addition not only enhances the connection between social media and subjective norms but also enriches our discussion on the mechanisms through which social media platforms can contribute to fostering a culture of environmental responsibility among consumers. Moreover, subjective norms developed from family, friends, and streamers have been stipulated to significantly influence intentions such as gifting in live-streaming contexts [27]. Thus, we propose the following hypothesis:

**H1b:**  *Consumers’ subjective norms towards transparent food production processes positively influence behavioral intentions.*

#### 2.1.4. Perceived Behavioral Control

Perceived behavioral control reflects user-perceived autonomy over engaging with the media, such as having time and finding value when watching food production videos. This component encompasses the perceived ease or difficulty of enacting a behavior and is informed by past experiences and anticipated impediments [19]. Perceived behavioral control, as posited by La Barbera and Ajzen [28], bolsters the salience of attitudes in the formulation of intentions. Evidence suggests that, in food supply chains, perceived behavioral control positively influences consumer behavior [29], and within social commerce, it has a positive effect on purchase intentions [30]. Therefore, we propose the following hypothesis:

**H1c:**  *Consumers’ perceived behavioral control of transparent food production processes positively influences their behavioral intentions.*

### 2.2. Perceived Trust (PT)

Perceived trust is the process of trust, emphasizing the importance of trust in the entire trust sequence [31] and is a very important factor for consumers when making shopping decisions. Cai et al. [32] indicate that the presence of short videos can influence consumers’ willingness to pay attention to products by stimulating their perceived value and trust. Through this process, viewers can gain a deeper understanding of the quality and production process of these foods, and further understand the food factory’s production standards and procedures. Lăzăroiu et al. [33] suggest that trust and perceived risk significantly influence consumer purchase decisions on social commerce platforms, with mobile payment services potentially influencing impulsive buying behavior. Based on the above, this study proposes the following hypothesis:

**H2:**  *Consumers’ perceived trust in transparent food production processes positively influences their behavioral intentions.*

### 2.3. Perceived Risk (PR)

Perceived risk, as evaluated by Hansen et al. [34], emerges as a critical determinant in consumer decision making, with propensities towards risk exerting an influence on behavioral intentions. Visual psychological imagery has been inversely associated with perceived risk; negative imagery tends to evoke adverse emotions and stress, amplifying perceived risk and dampening the inclination towards risky activities [35]. Heightened awareness and information-seeking behavior around food safety risks evoke increased consumer vigilance [36]. Thus, we propose the following hypothesis:

**H3:**  *Consumers’ perceived risk of transparent food production processes positively influences their behavioral intentions.*

### 2.4. Community Experience (CE)

As noted by Kim et al. [37], the online community experience is significantly influenced by the satisfaction of user needs, with information acquisition being paramount. Videos depicting food processes cater to an online community’s informational need. The enrichment of online knowledge communities via information technology bolsters consumer–business communication, where fostering an agreeable community experience is vital for maintaining user relationships [38]. Research indicates that community experience exerts a significant positive impact on brand image [39]. Customer experience during the service process is an important determinant of brand identification [40]. Therefore, we propose the following hypothesis:

**H4:**  *Consumers’ community experience with transparent food production processes positively influences brand identification.*

### 2.5. Brand Identification (Bi)

Brand relationships are formed through a series of consumer experiences and brand encounters [41]. Online knowledge community experiences indirectly affect purchase intentions for new products through group and brand identification, where user community experience is an important factor. Li et al. [42] indicated that user community experiences in online knowledge communities have a positive impact on brand identification, actively constructing and enhancing the experiential value of the community and providing users with the various experiences they need, thereby promoting them to spontaneously become brand advocates [43]. Zhao and Shi [39] found that brand image has a significant and positive impact on purchase intentions. Based on the above, this study proposes the following hypothesis:

**H5:**  *Consumers’ brand identification with transparent food production processes positively influences behavioral intentions.*

## 3. Research Methodology

### 3.1. Research Framework

**Figure 1:**
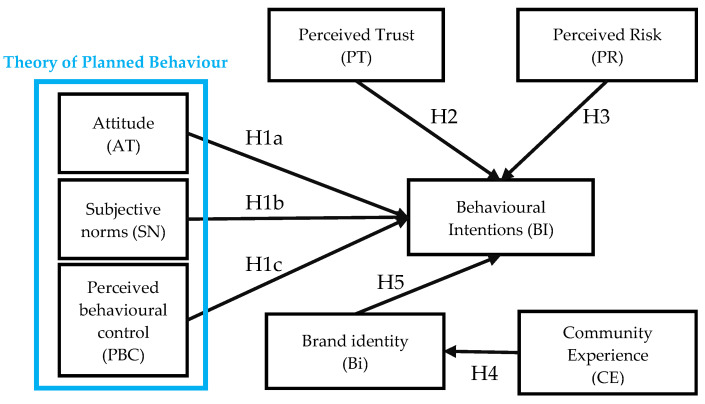
Conceptual framework and hypotheses of the study.

The research framework of this study integrates the core precepts of the Theory of Planned Behavior (TPB) with the addition of four supplementary constructs: ‘Perceived Trust’, ‘Perceived Risk’, ‘Brand Identification’, and ‘Community Experience’. This integration was intended to scrutinize the impact of food factory video presentations of their production processes on consumers’ purchase intentions. As Figure 1 shows, this comprehensive framework encapsulates the associations that exist between constructs, as informed by the literature review.

### 3.2. Research Questionnaire Design

The research questionnaire was meticulously structured into seven distinct sections, each crafted to measure specific constructs pertinent to the study’s objectives. This detailed design enhances the clarity and accuracy of data collection, facilitating a nuanced analysis of the factors influencing consumer behavior in the context of food safety.

The initial segment of the questionnaire was dedicated to assessing the theoretical framework based on the Theory of Planned Behavior (TPB). This section was adapted from Han et al. [44], encompassing 12 items divided equally into three core constructs: attitude towards behavior (four items), subjective norms (four items), and perceived behavioral control (four items). The selection and modification of these items were grounded in the need to tailor the theoretical constructs to the specific context of our study while preserving their conceptual integrity, ensuring that the measurements remained robust and relevant.

Subsequent sections were derived from recent and pertinent studies, carefully chosen to align with the current research objectives while ensuring that the constructs were measured with due reliability and validity.

(1) Perceived Trust: Modeled after Deng et al. [45], this section included three questions designed to capture respondents’ trust in food safety information.
(2) Perceived Risk: Following Li et al. [46], this section encompassed three questions that aimed to assess the perceived risks associated with food consumption.
(3) Community Experience: Drawing from Zhuo et al. [47], this domain was explored through two questions that investigated the influence of community experiences on food safety perceptions.
(4) Brand Identification: Adapted from Xue et al. [48], two questions were included to gauge how brand identification impacts consumer trust and behavior regarding food safety.
(5) Purchase Intention: Also following Xue et al. [48], this section comprised three questions aimed at assessing the intention to purchase safe food products.

By integrating these modified constructs into our questionnaire, we anchored our research to an existing empirical foundation, facilitating comparisons and discussions within the extant literature.

The final part of the questionnaire sought to gather essential demographic information from the participants, including sex, age, educational level, personal monthly income, occupation, and experience with food poisoning. These variables were included to enable a comprehensive analysis of how demographic factors might influence or correlate with the primary constructs of the study.

Except for the demographic data, all items were measured on a seven-point Likert scale ranging from strongly disagree (1) to strongly agree (7). This scale was chosen because of its ability to capture the intensity of respondents’ attitudes and perceptions, allowing for a nuanced understanding of the constructs being measured. The utilization of a seven-point scale also aligns with best practices in social science research, providing a balance between sensitivity and ease of response.

The operationalization of each construct through specifically designed questions was a critical process. This rigorous approach ensured that each measurement accurately captured the intended concept, thereby enhancing the reliability and validity of the collected data. Modifications from the source studies were judiciously made to align with the specific context of our research while upholding the conceptual and empirical rigor of the original constructs.

In summary, the design of our research questionnaire represents a comprehensive and methodologically sound approach to investigating the multifaceted factors influencing consumer behavior in the context of food safety. By adapting and integrating established theoretical frameworks and constructs, the questionnaire was poised to yield insights that were both reliable and deeply relevant to the study’s aims.

### 3.3. Sample and Data Collection

The study was meticulously conducted within the geographical bounds of Taiwan. This choice was underpinned by Taiwan’s vibrant consumer market and the significant adoption of digital marketing strategies, particularly relating to sustainable food purchasing behaviors. Taiwan’s context, marked by a substantial internet penetration rate and an emerging consciousness towards sustainable consumption practices among its populace, presented an academically fertile ground to investigate the impact of streaming video platform content on perceptions of food production processes.

To establish a rigorous framework for our investigation within the Taiwanese context, it was essential to precisely identify the population segment from which our sample was drawn. Our study zeroed in on Taiwan’s Generation Z, defined as the individuals born between 1997 and 2012. This demographic cohort forms a notable portion of the Taiwanese population, accounting for approximately 17% of the total, as indicated by the data from the National Department of Statistics. Given their intrinsic connection with digital media as ‘digital natives’ and their evolving consumer behaviors, focusing on this segment enabled us to gain meaningful insights into the patterns of sustainable food consumption influenced by digital content.

Additionally, an expressed interest in food, cooking, or nutrition, validated through responses in a preliminary screening survey, was mandatory for inclusion. This strategic focus on Generation Z was grounded in their growing influence as digital consumers and their potential to shape future trends in sustainable food consumption.

The questionnaire was disseminated following a convenience sampling strategy, particularly due to the practical challenges of engaging a highly specific subset of the population. The official questionnaire distribution spanned September to December 2023. Leveraging digital platforms prevalently used by the target demographic, such as Facebook, Instagram, and Line, alongside word-of-mouth campaigns, facilitated a broad yet targeted reach. Ethical guidelines were rigorously adhered to, with participant anonymity guaranteed, and the study’s purpose was communicated transparently at the outset of the questionnaire.

The integrity and accuracy of our findings necessitated a meticulously derived sample size. To this end, a preliminary power analysis was performed to ascertain the requisite sample size to ensure the statistical validity and reliability of our study. This procedure employed a variant of Cochran’s formula [49] for sample size determination, accounting for the specific parameters of our research context. The formula was as follows:

n_0_ = z^2^ p q/d^2^

where n_0_ represents the desired sample size for populations exceeding 10,000, z stands for the standard normal deviation (1.96 for a 95% confidence level); and p denotes the estimated proportion of the population to be included in the sample—here, approximately 17% of Taiwan’s Generation Z; q equals 1–0.17 (0.83), and d is the degree of accuracy desired, represented by a 5% margin of error.

Substituting the values into the Cochran formula yields

n_0_ = z^2^ p q/d^2^ = (1.96)^2^ (0.17) × (0.83)/(0.05)^2^ = 216

indicating that a sample of at least 216 participants was necessary to attain statistically significant results. Our aim was to collect data from a minimum of 250 respondents to accommodate potential dropouts and to ensure the validity of the findings. This strategic approach enabled us to mitigate the impact of potential data loss owing to invalid responses or attrition. Consequently, we successfully gathered 226 valid questionnaires, thereby exceeding the threshold required for statistical significance and ensuring the robustness of the research outcomes. Notably, the sample displayed a predilection towards female participants, a phenomenon consistent with prevailing trends in consumer research, particularly within food-related studies where females disproportionately represent the main purchasers of food for households [50]. This gender bias, while reflective of societal roles, warrants consideration when interpreting the findings, as it may influence the generalizability of the study’s conclusions.

This methodological rigor underscores our commitment to achieving a high level of accuracy and reliability in examining sustainable food consumption trends among Taiwan’s Generation Z, as influenced by digital content. Through careful planning and execution, our study adheres to established statistical standards and provides meaningful insights into the behaviors and preferences of a crucial demographic segment, thereby contributing to the broader discourse on sustainable consumption practices.

Demographic insights (detailed in Table 1) revealed substantial engagement with food processing content among participants, with 73% endorsing the regular viewing of such content online. Interest levels varied across content types, with the highest preference for videos showcasing innovative cooking techniques (65%), closely followed by cultural or traditional cooking methods (55%) and sustainable or ethical food processing practices (45%).

### 3.4. Methods of Data Analysis

In light of the calculated sample size and aiming to uphold the highest standards of statistical integrity, we used IBM SPSS Statistics 27 and AMOS 28 for our data analysis. The rationale behind leveraging Maximum Likelihood Estimation through structural equation modeling was its suitability for the sample size confirmed against Cochran’s formula, acknowledging that this method provides reliable estimates and inferential statistics for sample sizes akin to ours. This approach allowed us to thoroughly test our theoretical model’s fit and the validity of our research hypotheses with the necessary empirical rigor needed for conclusive insights. The analysis comprised descriptive statistical measures including frequency distributions, means, and standard deviations. It also confirmed the reliability and validity of the instruments, followed by the application of a Maximum Likelihood Estimation via structural equation modeling. These analyses collectively sought to substantiate the theoretical model’s fit and test the validity of the research hypotheses with empirical rigor.

## 4. Analysis and Results

### 4.1. Measurement Model: Reliability and Validity

The reliability and validity assertions for the constructs within the study were ascertained, and are presented in Table 2. For reliability, Cronbach’s alpha values above 0.7 are denoted as high reliability as per Nunnally [51]. In this study, all constructs displayed Cronbach’s alpha values exceeding the 0.7 threshold, which denotes a high level of internal consistency for the measures employed.

Concurrently, convergent validity was assessed, and Fornell and Larcker [52] stipulated benchmarks for this validation criterion, including standardized factor loadings above 0.5, average variance extracted (AVE) surpassing 0.5, and composite reliability (CR) values exceeding 0.6. Additionally, Bagozzi and Yi [53] posit that a CR value above 0.60 signifies commendable internal consistency. The factor loadings, AVE, and CR results presented in Table 2 surpassed the specified standards, thereby affirming both convergent validity and internal consistency of the constructs within the study.

Discriminant validity was appraised by adhering to Fornell and Larcker’s [52] guidelines, which recommend that the square root of the average variance extracted (AVE) for each construct should exceed the corresponding inter-construct correlation coefficients. Table 3 shows that all constructs met this condition, thereby establishing discriminant validity and illustrating that the measurement model sufficiently distinguished between the constructs.

### 4.2. Model Fit Test

We examined the proposed hypotheses using the Maximum Likelihood Estimation (MLE) method. The ratio x2/df was 3.618, implying a fairly good model fit. The CFI, TLI, and IFI values were 0.935, 0.905, and 0.937, respectively, suggesting acceptable fit. The RMSEA value was 0.083, which was within the acceptable range. Although the GFI (0.871) and AGFI (0.779) values were slightly below the standard criteria, Bagozzi and Yi [53] suggested that a GFI above 0.90, a conservative threshold, and a value exceeding 0.80 are considered acceptable. Considering that most of the fitness indices satisfied or surpassed the set benchmarks, the model from this investigation exhibited a strong correlation with the data, thereby demonstrating its superior conformity.

### 4.3. Overall Model Path Analysis

**Figure 2:**
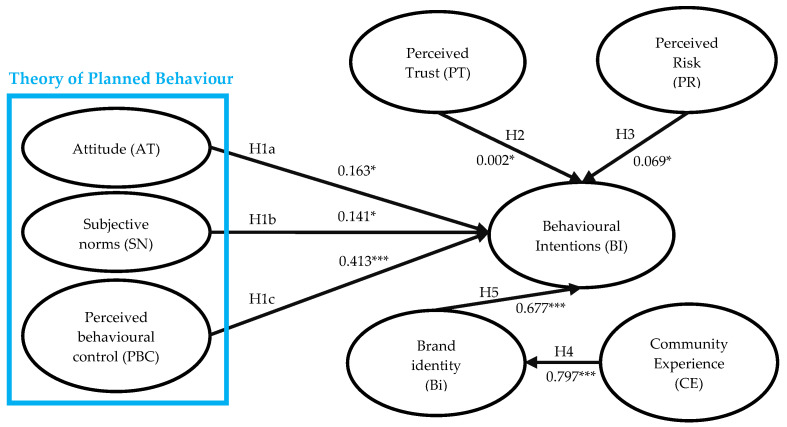
Structural Model Analysis Diagram. Note: * *p* < 0.05, *** *p* < 0.001.

The structural model and hypothesized relationships were analyzed using Structural Equation Modeling (SEM), with the path relationships delineated in Figure 2. The path coefficients indicate significant relationships: H1a through H1c depict attitudinal constructs and their impact on behavioral intention, all with *p*-values less than 0.05. The analysis revealed a strong effect of perceived behavioral control (H1c) on behavioral intention, with the largest standardized beta coefficient (β = 0.647) and a high statistical significance (*p* < 0.001).

Furthermore, consumer trust (H2) has a notable influence on behavioral intention, mirroring the results of attitudes and subjective norms. Upon reevaluation of the statistical analysis, it was acknowledged that the coefficient indicating the influence of consumer trust (H2) on behavioral intention was 0.002. This numerical value suggests that, while the direction of influence is positive, the magnitude of this effect is minimal and may not be as substantial as previously indicated. Therefore, based on the current dataset, it is more accurate to state that consumer trust has a limited and possibly negligible practical influence on behavioral intention. Interestingly, perceived risk (H3) has a comparatively less yet statistically significant negative impact on intention (β = 0.092, *p* < 0.05). Finally, community experience (H4) has a profound effect on brand identification (β = 0.717, *p* < 0.001), and brand identification (H5) impacts behavioral intention with a similar substantial coefficient (β = 0.646, *p* < 0.001).

Table 4 consolidates the path analysis outputs, reinforcing that Hypotheses H1a, H1b, H1c, H2, H3, H4, and H5 have all been empirically supported, indicating that the theoretical framework is well-substantiated and the hypothesized paths in the model are affirmed, forming a robust basis for drawing informed conclusions from the study’s results.

## 5. Discussion

The findings of this investigation resonate with established behavioral and social psychological paradigms that postulate that consumer attitudes are pivotal in molding behavioral intentions. Corroborating Wolf et al. [20], the positive relationship between consumer attitudes and behavioral intentions underscores the pivotal role that perceptions of transparent food production processes play in shaping consumer purchasing decisions. This finding indicates that viewing transparent processes on streaming platforms amplifies positive consumer perceptions, thus fortifying the likelihood of purchasing.

Extending the discourse on social influence, the demonstrated positive effect of subjective norms on behavioral intentions echoes Joo et al. [23], fortifying the premise that the support of influential others towards transparent practices in food production amplifies consumers’ propensity to purchase. This finding highlights the importance of social endorsements and peer perspectives in the decision-making processes.

Parallel to La Barbera and Ajzen [28], the present study illuminates the significant predictive power of perceived behavioral control over behavioral intentions. This indicates that, when consumers perceive a greater degree of control facilitated by their capability to access informative content about food production, their purchase intentions are reinforced.

The integration of the constructs of perceived trust and perceived risk substantiates the multidimensional nature of consumer decision making. According to Cai et al. [32], perceived trust garners a substantive positive axis with behavioral intentions, thereby implying that building a trustworthy image through transparent communication channels is crucial for influencing consumer choices. Conversely, aligned with Sobkow et al. [35] and Guo et al. [54], the positive sign associated with perceived risk contradicts the conventional wisdom. A positive coefficient suggests that consumers who acknowledge the risks associated with food consumption may be more thoroughly engaged and conscientious, thus motivating the intention to indulge in well-informed purchases.

In examining community experience and brand identification, this investigation draws parallels with the existing literature [39,40,55], substantiating that consumers’ interactions within brand communities are instrumental in fostering a strong sense of brand identification, which in turn feeds into their intentional learning. This finding suggests that immersive and engaging community experiences are critical in cultivating brand affinity and loyalty.

Finally, this study’s exploration of the link between brand identification and behavioral intentions supports the edifice established by Zhao and Shi [39], Li et al. [42], and Fazli-Salehi et al. [56]. This provides empirical support for the assertion that, when consumers identify with a brand’s values, especially those associated with transparency and ethical practices, they are more predisposed to purchase.

This study provides robust evidence of the influence of transparent food production process videos on streaming services that attract Generation Z consumers. This demonstrates a marked trend towards the purchase of these products, which is a direct result of the clarity and openness of these videos. This study systematically confirms the intricate relationship between social influence, perceived control, trust, and brand identity, providing valuable insights for food-related businesses seeking to refine their marketing strategies to resonate with savvy consumers’ demographics.

From a managerial perspective, this study’s findings underscore the significant influence of customer attitudes, social norms, and the degree to which consumers feel control over their purchase intentions. These insights suggest that by effectively utilizing their official online platforms such as social media and websites, food manufacturers can transcend traditional informational roles to become instrumental tools in fostering trust and captivating consumer engagement. This approach provides a dynamic two-way communication channel for consumer inquiries and feedback, making these platforms valuable tools for establishing brand loyalty.

Nevertheless, these opportunities have heightened the responsibilities of content management. Transparency that attracts consumers must be managed with a deft hand to avoid revealing the unpalatable aspects of production that may deter consumers. Therefore, food businesses must maintain a delicate balance between education and illumination, without generating adverse reactions. It is imperative to craft messages that enhance consumers’ learning experiences without compromising their brand perception.

Moreover, this study revealed the critical role of active community involvement in brand-building activities. In the digital sphere, communal experiences have a profound impact on shaping brand identities and driving consumer behavior. The findings suggest that modern consumers, particularly within Generation Z, share online experiences, and the sense of collective endorsement they bestow are increasingly influential factors in their decision-making process. Therefore, food businesses should consider integrating community-focused initiatives into their marketing strategies to forge stronger connections and more positive food-purchasing patterns.

## 6. Conclusions

The empirical evidence gleaned from this investigation provides a nuanced understanding of the modern consumer psyche, particularly in relation to the transparency in food production. This section synthesizes the key findings of the study, notes its inherent limitations, and presents a course for subsequent inquiries.

### 6.1. Research Conclusions

This study meticulously analyzed the pertinent transparency factors listed in Table 2, revealing that food processing videos on streaming platforms markedly heightened consumer awareness regarding the transparency of food production processes. This revelation is of paramount importance for forging trust in the safety and quality of food products, which is instrumental in influencing consumers’ purchase intentions. Furthermore, trust acts as a critical conduit for effectuating tangible shifts in consumer behavior towards making informed purchasing decisions. Moreover, examining the data related to the influence of news outlets, newspapers, magazines, and international trends on the consumption of publicly available food processing videos, it becomes apparent that consumers, particularly Generation Z, are inclined towards leveraging social media platforms to acquire information about food safety and quality. This underscores the profound impact of external media sources and prevailing trends on consumer purchasing decisions and food consumption patterns.

Additionally, by employing the theory as an analytic scaffold, this study elucidates how variables such as perceived trust, perceived risk, community experience, and brand identity play pivotal roles in mediating consumers’ receptivity towards food production videos. These variables not only influence the acceptance of such informational content but also significantly shape consumers’ behavioral intentions towards food purchasing decisions. The collective findings of this study underscore the criticality of transparency in the food manufacturing sector. They offer substantive insights to industry stakeholders to refine their marketing approaches by incorporating transparency-centric strategies. This initiative will not only augment consumers’ comprehension of food production intricacies, but will also play a crucial role in fostering a more informed and health-conscious food consumption culture.

The culmination of this research firmly supports the hypothesis that a transparent portrayal of food manufacturing processes via streaming platforms engenders a positive recalibration of consumer attitudes towards food products. This attitudinal shift is seminal in navigating consumers’ journey from mere viewership to active purchasing, wherein factors such as perceived risk, trust, and brand affiliation become significantly pronounced. A notable inference drawn from these findings is the imperative need for food manufacturers to reconceptualize transparency not as an optional attribute, but as a strategic asset indispensable for brand differentiation and cultivating consumer trust. Remarkably, the empirical evidence shows conspicuous engagement of consumers with transparent production narratives, suggesting that such content is not merely informative but serves as a potent catalyst in transforming viewers into a dedicated and trustworthy customer base. Collectively, these insights provide a transformative paradigm in food marketing strategies, underscoring the indispensability of transparency in nurturing a trusting relationship with consumers and steering the food industry towards a future characterized by informed choices and sustainable consumption patterns.

### 6.2. Research Limitations and Future Research Directions

Despite these insights, it is important to acknowledge the limitations of this study. The primary limitation is the concentration of Generation Z as the target demographic, which may not be generalizable across the various age groups. Future research may benefit from a broader demographic lens to capture the full spectrum of behaviors related to streaming platform content. Moreover, the limited responses concerning food poisoning experiences require an enlarged sample size and further investigation of this dimension, as this could offer valuable insights into risk perception and information scrutiny. However, in this study, the actual impact of perceived trust (H2) on behavioral intention was limited and negligible. Therefore, future research should explore the relationship between perceived trust and behavioral intention, which consists of two structural components: normative beliefs and the motivation to comply. However, in this study, normative beliefs were primarily assessed when measuring subjective norms, and there was insufficient measurement of motivation to comply. This could potentially affect the influence of subjective norms on behavioral intentions. Therefore, future research should consider more comprehensive SN measurements.

Finally, cultural contexts undeniably shape consumer attitudes and behaviors. Although this study is one of the first few stepping stones, the variability inherent in global consumer bases poses both a challenge and an opportunity for international brands to optimize their transparency strategies. Subsequent research must consider the intricate interplay between cultural norms and transparency perceptions, and tailor communication strategies that not only inform but also resonate with diverse audiences.

This study underscores the significance of transparency in food production processes as a catalyst for securing consumer trust and loyalty in an era requiring corporate openness. The move towards transparent practices, facilitated by digital platforms, is not a fleeting trend but a paradigm shift signifying a new epoch of consumer–brand relationships based on openness, dialogue, and mutual trust.

## Figures and Tables

**Table 1:** Demographic analysis examining the characteristics of a population.

*N* = 226	Item	Population	Percentage (%)
Have you ever experienced food poisoning	Yes	38	16.8
	No	188	83.2
Gender	Male	78	34.5
	Female	148	65.5
Age	20 years and below	46	20.4
	21–30 years	180	79.6
Level of Education	High school/vocational or below	12	5.3
	College/university	180	79.6
	Master’s or above	34	15.0
Monthly personal income	Less than NTD 20,000 (USD 660) (inclusive)	166	73.5
	NTD 20,001–40,000 (USD 660–1320)	52	23.0
	NTD 40,001–60,000 (USD 1320–1980)	6	2.7
	NTD 60,001–80,000 (USD 1980–2640)	0	0.0
	Above NTD 80,001 (USD 2640)	2	0.9
Occupation	Student	182	80.5
	Army, civil service, and education	8	3.5
	Service industry	10	4.4
	Freelance	6	2.7
	Traditional manufacturing	2	0.9
	Specialized occupation (e.g., doctor and lawyer)	4	1.8
	Other	14	6.2

**Table 2:** Results for the factor loading, reliability, and validity.

Variables/Items	Mean	Standard Deviation	Standardized Factor Loading	AVE	CR	Cronbach’s α
Attitude (AT)	5.918	0.848		0.559	0.834	0.830
1. The disclosure of food processing videos by food providers can reassure consumers about consuming their products	5.820	1.052	0.810 ***			
2. The disclosure of food processing videos by food providers can help consumers understand the food production process better	6.210	0.984	0.714 ***			
3. The disclosure of food processing videos by food providers can convey food safety messages to consumers	5.770	1.139	0.818 ***			
4.The disclosure of food processing videos by food providers is important to consumers	5.870	0.984	0.634 ***			
Subjective norms (SN)	4.945	1.014		0.571	0.841	0.836
5. My family thinks I should choose products with disclosed food processing videos	4.580	1.422	0.840 ***			
6. My friends/colleagues believe that one should choose products with disclosed food processing videos to ensure product quality	4.620	1.315	0.825 ***			
7. I am influenced by news, newspapers, and magazines to purchase products with disclosed food processing videos	5.230	1.033	0.658 ***			
8. I am influenced by international trends to purchase products with disclosed food processing videos	5.350	1.146	0.683 ***			
Perceived behavioral control (PBC)	5.044	1.049		0.596	0.853	0.840
9. I am willing to pay extra for food safety for products that have disclosed food processing videos	4.440	1.472	0.599 ***			
10. I believe that products with disclosed food processing videos are more likely to improve product quality	5.430	1.130	0.839 ***			
11. When dining, I will choose products with disclosed food processing videos	4.890	1.349	0.837 ***			
12. I feel that choosing products with disclosed food processing videos is the right choice	5.410	1.121	0.788 ***			
Perceived trust (PT)	5.513	1.012		0.707	0.878	0.869
13. The disclosure of food processing videos by food providers can offer detailed product information	5.560	1.139	0.820 ***			
14. The disclosure of food processing videos by food providers allows for an easy comparison of ingredients across similar products	5.450	1.123	0.919 ***			
15. I can quickly obtain product information through disclosed food processing videos	5.530	1.148	0.778 ***			
Perceived risk (PR)	3.613	1.385		0.587	0.809	0.803
16. I may have to spend a lot of time getting used to products with disclosed food processing videos	3.550	1.538	0.796 ***			
17. I worry that products with disclosed food processing videos may cause me psychological discomfort	3.370	1.682	0.821 ***			
18. I am concerned that products with disclosed food processing videos may not effectively address the food safety issues I face	3.920	1.682	0.674 ***			
Community experience (CE)	5.509	0.924		0.744	0.853	0.845
19. I can get some useful information or resources from the food processing videos that are disclosed	5.620	0.917	0.905 ***			
20. I can provide the information that others need for the food processing videos that are disclosed	5.400	1.063	0.818 ***			
Brand identity (Bi)	5.460	0.962		0.682	0.811	0.810
21. I trust the brands of products that have disclosed food processing videos	5.290	1.039	0.854 ***			
22. Disclosure of food processing videos is an honest brand strategy	5.630	1.060	0.797 ***			
Behavioral intention (BI)	5.451	0.922		0.766	0.908	0.905
23. I am interested in purchasing brand food products with disclosed food processing videos	5.460	1.042	0.892 ***			
24. Overall, I am satisfied with brand food products that have disclosed food processing videos	5.380	0.974	0.826 ***			
25. I am considering purchasing brand food products with disclosed food processing videos	5.510	0.999	0.906 ***			

Note 1: CR: composite reliability; AVE: average variance extracted. Note 2: *** *p* < 0.001.

**Table 3:** Correlation coefficients of the measurement model and the square root of AVE.

	1	2	3	4	5	6	7	8
1. AT	**0.748**							
2. SN	0.286 **	**0.756**						
3. PBC	0.321 **	0.698 **	**0.772**					
4. PT	0.434 **	0.469 **	0.562 **	**0.841**				
5. PR	−0.231 **	0.210 **	0.250 **	0.140 **	**0.766**			
6. CE	0.391 **	0.396 **	0.483 **	0.582 **	−0.011	**0.863**		
7. Bi	0.480 **	0.399 **	0.476 **	0.610 **	−0.084	0.646 **	**0.826**	
8. BI	0.465 **	0.552 **	0.647 **	0.574 **	0.092	0.637 **	0.717 **	**0.875**

Note 1: The values on the diagonal (bold font on the slash) represent the square root of the Average Variance Extracted (AVE) of the latent variables. Note 2: AT: attitude; SN: subjective norms; PBC: perceived behavioral control; PT: perceived trust; PR: perceived risk; CE: community experience; Bi: brand identity; BI: behavioral intention. Note 3: ** *p* < 0.01.

**Table 4:** Results of the path analysis and confirmation of hypotheses.

Hypothesized Paths	Unstandardized Coefficient	Standardized Coefficients	S.E.	C.R.	*p*	Verification Results
H1a:AT → BI	0.205	0.163	0.086	2.370	*	Supported
H1b:SN → BI	0.157	0.141	0.082	1.919	*	Supported
H1c:PBC → BI	0.346	0.413	0.073	4.715	***	Supported
H2:PT → BI	0.002	0.002	0.059	0.029	*	Supported
H3:PR → BI	0.040	0.069	0.037	1.093	*	Supported
H4:CE → Bi	0.860	0.797	0.081	10.614	***	Supported
H5:Bi → BI	0.574	0.677	0.075	7.637	***	Supported

Note 1: AT: attitude; SN: subjective norms; PBC: perceived behavior control; PT: perceived trust; PR: perceived risk; CE: community experience; Bi: brand identity; BI: behavioral intention. Note 2: * *p* < 0.05, *** *p* < 0.001.

## Data Availability

The original contributions presented in the study are included in the article, further inquiries can be directed to the corresponding author.
